# Predominant role of interferon-γ in the host protective effect of CD8^+^ T cells against *Neospora caninum* infection

**DOI:** 10.1038/srep14913

**Published:** 2015-10-09

**Authors:** Alexandra Correia, Pedro Ferreirinha, Sofia Botelho, Ana Belinha, Catarina Leitão, Íris Caramalho, Luzia Teixeira, África González-Fernandéz, Rui Appelberg, Manuel Vilanova

**Affiliations:** 1 Instituto de Investigação e Inovação em Saúde, Universidade do Porto, and IBMC - Instituto de Biologia Molecular e Celular, Universidade do Porto, 4150-180 Porto, Portugal; 2 ICBAS - Instituto de Ciências Biomédicas de Abel Salazar, Universidade do Porto, 4050-313 Porto, Portugal; 3 Instituto de Medicina Molecular, Faculdade de Medicina, Universidade de Lisboa, 1649-028 Lisboa, Portugal; 4 UMIB-Unidade Multidisciplinar de Investigação Biomédica, Universidade do Porto, Porto; 5 Inmunología, Centro de Investigaciones Biomédicas (CINBIO), Instituto de Investigación Biomédica, Universidade de Vigo, Campus Lagoas Marcosende, E-36200 Vigo, Spain

## Abstract

It is well established that CD8^+^ T cells play an important role in protective immunity against protozoan infections. However, their role in the course of *Neospora caninum* infection has not been fully elucidated. Here we report that CD8-deficient mice infected with *N. caninum* presented higher parasitic loads in the brain and lungs and lower spleen and brain immunity-related GTPases than their wild-type counterparts. Moreover, adoptive transfer of splenic CD8^+^ T cells sorted from *N. caninum*-primed immunosufficient C57BL/10 ScSn mice prolonged the survival of infected IL-12-unresponsive C57BL/10 ScCr recipients. In both C57BL/6 and C57BL/10 ScSn mice CD8^+^ T cells are activated and produce interferon-γ (IFN-γ) upon challenged with *N. caninum.* The host protective role of IFN-γ produced by CD8^+^ T cells was confirmed in *N. caninum*-infected RAG2-deficient mice reconstituted with CD8^+^ T cells obtained from either IFN-γ-deficient or wild-type donors. Mice receiving IFN-γ-expressing CD8^+^ T cells presented lower parasitic burdens than counterparts having IFN-γ-deficient CD8^+^ T cells. Moreover, we observed that *N. caninum*-infected perforin-deficient mice presented parasitic burdens similar to those of infected wild-type controls. Altogether these results demonstrate that production of IFN-γ is a predominant protective mechanism conferred by CD8^+^ T cells in the course of neosporosis.

*Neospora caninum* is a cyst-forming coccidian parasite responsible for clinical infections in a wide range of animal hosts including bovines^1^. In cattle *N. caninum* is a major cause of abortions and stillbirths occurring worldwide thus having a major economic impact on dairy industry^2^. Currently, no effective commercially available vaccine exists against neosporosis^3^. Therefore, a better understanding of immune mechanisms mediating host resistance to this infectious disease may be helpful in designing immune-mediated preventive approaches for neosporosis.

Studies performed in mice and cattle infected with *N. caninum* have shown that dendritic cells and macrophages^4^,^5^,^6^, NK cells^7^,^8^ and CD4^+^ T cells^9^,^10^,^11^ provide different effector functions in protective immunity to neosporosis. As *N. caninum* is an obligate intracellular parasite, it could also be expected that CD8^+^ T cells participate in host protection against this parasite^12^ as it has previously been shown in mice infected with *Toxoplasma gondii, a* closely related pathogen^13^. Indeed, a study in which CD8^+^ T cells were depleted using a specific monoclonal antibody (mAb) revealed a mild protective effect of this lymphocyte population in *N. caninum* infected mice^9^. Nevertheless, the underlying mechanisms responsible for this protection remain poorly defined. Moreover, another study indicated that these cells could also exacerbate the neurologic symptoms resulting from *N. caninum* infection^14^. Therefore, a reassessment of the role that these cells may play in *N. caninum* infection is needed. In this study we directly addressed the role of CD8^+^ T cells in the course of experimental murine neosporosis. Using different murine models, we confirmed that CD8^+^ T cells have a protective role in *N. caninum* infected hosts and provide compelling evidence showing that production of IFN-γ rather than cytotoxic function mediates their immunoprotective role.

## Results

### CD8^+^ T cells are expanded and activated in *N. caninum*-infected C57BL/6 mice

**Figure 1:**
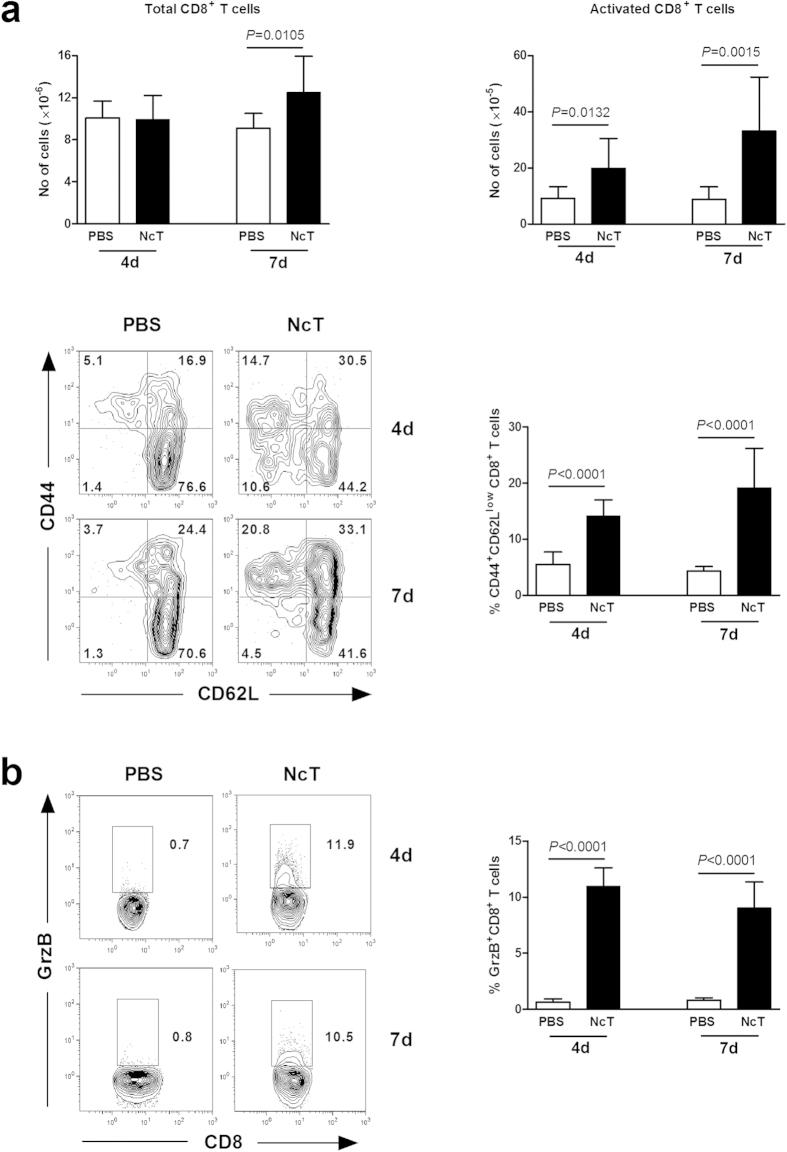
CD8^+^ T cells are activated expand and differentiate upon *N. caninum* infection. (**a**) Numbers of total and activated (CD44^+^CD62L^low^) CD8^+^ T cells, and percentage of activated CD8^+^ T cells, as indicated, and (**b**) percentage of splenic granzyme B^+^ (GrzB) cells of 4- and 7-day infected mice (NcT) and sham-infected controls (PBS). Bars represent means plus one SD of pooled data from three independent experiments (n = 9 for controls, n = 11 for 4-day infected mice and n = 13 for 7-day infected mice). Unpaired two-tailed *t*-test was used to compare parasite-inoculated vs respective control mouse groups. Statistical significance between infected mice and controls is indicated above bars. Contour plots correspond to a representative example of CD8-gated T cells of the analysed samples. Quadrants and regions were set according to isotype control-stained samples. Numbers within contour plots correspond to the percentage of cells in each quadrant or region.

It has been extensively shown that CD8^+^ T cells are important in host protection against intracellular protozoan parasites^15^. Here, we used wild-type (WT) C57BL/6 (B6) mice, which are susceptible to chronic neosporosis but resist acute infection^16^, to determine whether CD8^+^ T cells are activated in the course of acute *N. caninum* infection, established by i.p. injection of 1 × 10^7^ *N. caninum* tachyzoites (NcT). Sham-infected controls were similarly treated with PBS alone. As shown in Fig. 1a, higher numbers and frequencies of CD8^+^ T cells with a CD44^+^CD62L^low^ surface phenotype, indicative of cell activation^17^,^18^,^19^, were observed in the spleen of infected mice as compared to controls, 4 and 7 days upon the parasitic challenge. Moreover, higher proportions of granzyme B^+^ CD8^+^ T cells were also detected in the spleen of the infected mice, indicative of Cytotoxic T Lymphocyte (CTL) differentiation (Fig. 1b)^20^. In accordance with the above results, increased total CD8^+^ T cell numbers were observed in the spleen of *N. caninum*-infected mice by 7 days of infection (Fig. 1a). Altogether, these results show that CD8^+^ cells are activated and expanded in the course of *N. caninum* infection. In the infected mice splenic CD4^+^ T cells were also found expanded and similarly displayed an activated phenotype (Supplementary Fig. S1).

### CD8-deficient mice are more susceptible to *N. caninum* infection than wild-type controls

**Figure 2:**
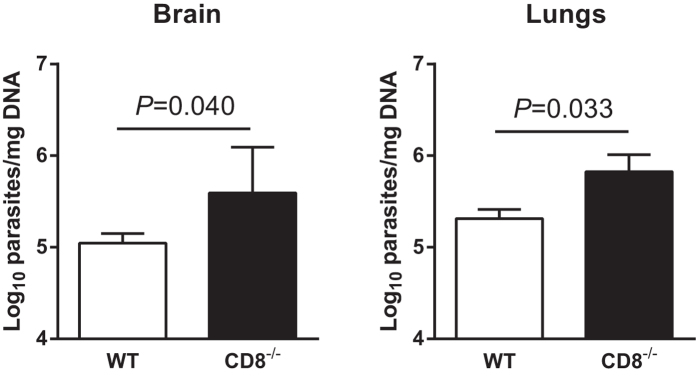
Increased susceptibility to *N. caninum* infection in *CD8a*^*−*/*−*^ mice. Parasitic load of brain and lungs tissue assessed by qPCR specific for *N. caninum* DNA in WT or *CD8a*^*−*/*−*^ mice, as indicated, 7 days after i.p. inoculation of 1 × 10^7^ NcT. Bars represent means plus one SD of pooled data from two independent experiments (n = 10 for controls and n = 12 for infected mice). Unpaired two-tailed *t*-test was used to compare parasite-inoculated vs respective control mouse groups. Statistical significance between infected mice and controls is indicated above bars.

Having ascertained that CD8^+^ T cells were activated in *N. caninum* infected B6 mice, we assessed by quantitative real time PCR (qPCR) specific for *N. caninum* DNA the parasitic load in the brain and lungs of CD8-deficient (*Cd8a*^*−/−*^) mice and WT controls, 7 days upon i.p. inoculation with 1 × 10^7^ NcT. As shown in Fig. 2, significantly higher parasitic DNA levels were detected in both organs of *Cd8a*^*−*/*−*^ mice than in those of WT controls. WT and *Cd8a*^*−/−*^ mice survived for at least 40 days upon the parasitic challenge without evidencing clinical signs. At this time-point parasitic burden was lower than the one detected for the respective groups 7 days upon infection. Nevertheless, *Cd8a*^*−/−*^ mice still presented a higher parasitic load in the brain than the WT controls (Supplementary Fig. S2).These results altogether indicate that CD8^+^ T cells have a host-protective role in the course of *N. caninum* infection.

### Transfer of CD8^+^ T cells isolated from infected C57BL/10 ScSn mice prolongs survival of *N. caninum*-infected C57BL/10 ScCr mice

**Figure 3:**
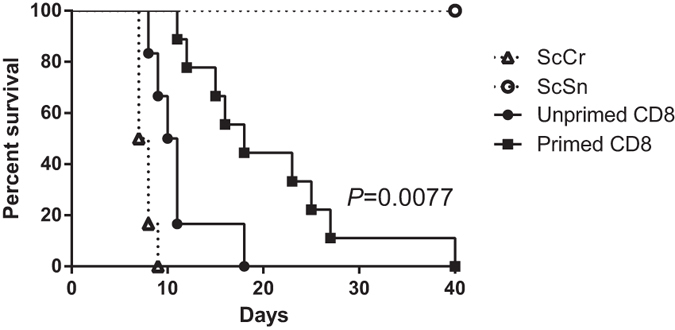
Transfer of primed CD8^+^ T cells prolongs survival of *N. caninum*-challenged ScCr mice. Survival of ScCr mice infected with 5 × 10^5^ NcT 16 h upon the adoptive transfer of CD8^+^ T cells obtained from the spleen of ScSn mice injected with PBS (Unprimed CD8) or infected with 1 × 10^7^ NcT (Primed CD8). Survival of similarly infected ScCr and ScSn non-transferred controls are also shown, as indicated. Statistical difference between the two transferred groups was calculated with the log-Rank test (n = 6, control; n = 9, infected) and is indicated. Statistical differences between the unprimed CD8 and primed CD8 groups and ScCr controls were of *P *= 0.0061 and *P *< 0.0001, respectively. Data correspond to pooled results of two independent experiments.

Since *Cd8a*^*−*/*−*^ mice presented higher susceptibility to *N. caninum* infection than their WT counterparts, CD8^+^ T cells are likely able to provide immune protection against this parasite infection. We thus asked whether CD8^+^ T cells from immunosufficient C57BL/10 ScSn (ScSn) mice could protect congenic C57BL/10 ScCr (ScCr) immunodeficient mice, unresponsive to IL-12 21, which have a deficient immune response to *N. caninum*^22^. As observed in B6 mice, higher proportions of splenic CD8^+^ T cells displaying an activated phenotype (CD44^+^CD62L^low^) were detected in infected ScSn mice than in sham-infected controls (Supplementary Fig. S3). Having determined their activated status, splenic CD8^+^ T cells were purified by flow cytometry sorting from i.p. NcT-infected and PBS treated ScSn mice. 1 × 10^6^ sorted cells were then transferred by intravenous injection into ScCr mice that were i.p. infected with 5 × 10^5^ NcT 16 h after the adoptive transfer. As shown in Fig. 3, mice that received CD8^+^ T cells from infected *N. caninum*-resistant donors survived longer than recipients transferred with CD8^+^ T cells sorted from sham-infected donors or than non-transferred ScCr controls. Curiously, a slight protective effect was also observed in mice receiving unprimed CD8^+^ T cells. As expected, all ScSn mice survived the parasitic challenge. This result is indicative that *in vivo* primed CD8^+^ T cells have a protective effect against *N. caninum* infection. However, CD8^+^ T cell-dependent immunity on its own could not confer full protection in a mouse lacking IL-12 signalling which also affects CD4^+^ T cells and NK cells.

### Limited effect of perforin expression in the host protective role of CD8^+^ T cells

**Figure 4:**
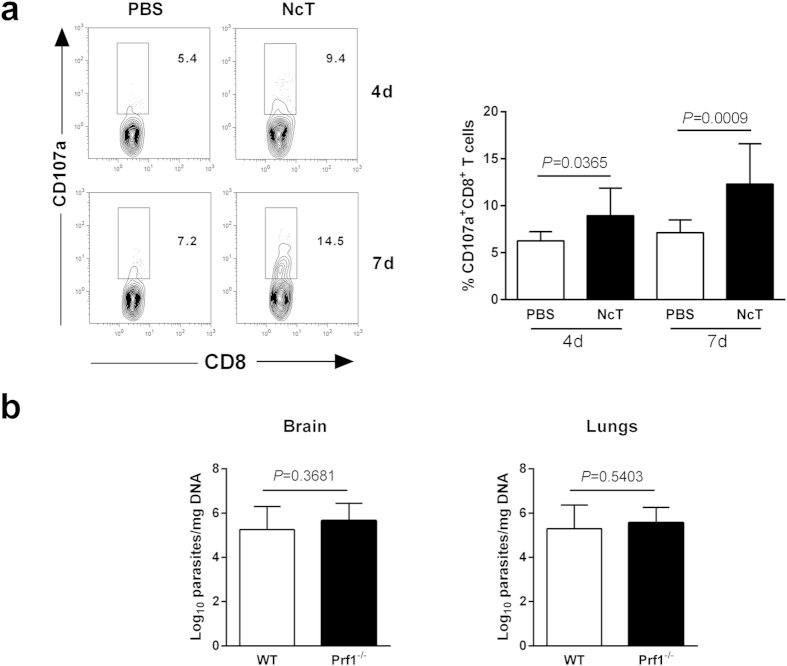
Perforin-deficiency do not increase the susceptibility to acute *N. caninum* infection. (**a**) Percentage of CD107a^+^ cells on total CD8^+^ T cells detected in the spleen of infected mice and controls. Bars represent means plus one SD of pooled data from two independent experiments (n = 6 and n = 10 for 4- and 7-day controls, respectively, and n = 10 and n = 12 for 4- and 7-day infected mice, respectively). Unpaired two-tailed *t*-test was used to compare parasite-inoculated vs respective control mouse groups. Statistical significance between infected mice and controls is indicated above bars. Contour plots correspond to a representative example of the analysed samples. Analysis regions were set according to isotype control-stained samples. Numbers within contour plots correspond to the percentage of cells in the analysis region shown. (**b**) Parasitic load of brain and lung tissue assessed by qPCR specific for *N. caninum* DNA in WT or *Prf1*^*−*/*−*^ mice, as indicated, 7 days after i.p. inoculation of 1 × 10^7^ NcT. Bars represent the mean plus one SD of pooled data from two independent experiments (n = 10 per group).

Expression of surface CD107a (LAMP-1) has been shown to be a marker for cytotoxic CD8^+^ T-cell activity. This expression is associated with loss of perforin following T cell stimulation by antigen^23^. Therefore, CD107a expression was assessed on the surface of splenic CD8^+^ T cells of B6 mice 4 and 7 days upon infection with *N. caninum* and compared with control animals. As shown in Fig. 4a, higher proportions of CD107a-expressing CD8^+^ T cells were found in the infected mice, indicating that degranulation was induced in these cells. Therefore, to assess whether perforin-dependent cytotoxicity could be protective against *N. caninum* infection, perforin-deficient (*Prf1*^*−*/*−*^) mice and WT B6 controls were i.p. infected with 1 × 10^7^ NcT and the parasitic burden evaluated in the brain and lungs. As shown in Fig. 4b, no statistically significant difference in parasitic burden was observed between the two infected groups. These results indicate that perforin-mediated cytotoxicity is not required for protection against an acute *N. caninum* infection.

### Production of IFN-γ mediates the protective effect of CD8^+^ T cells

**Figure 5:**
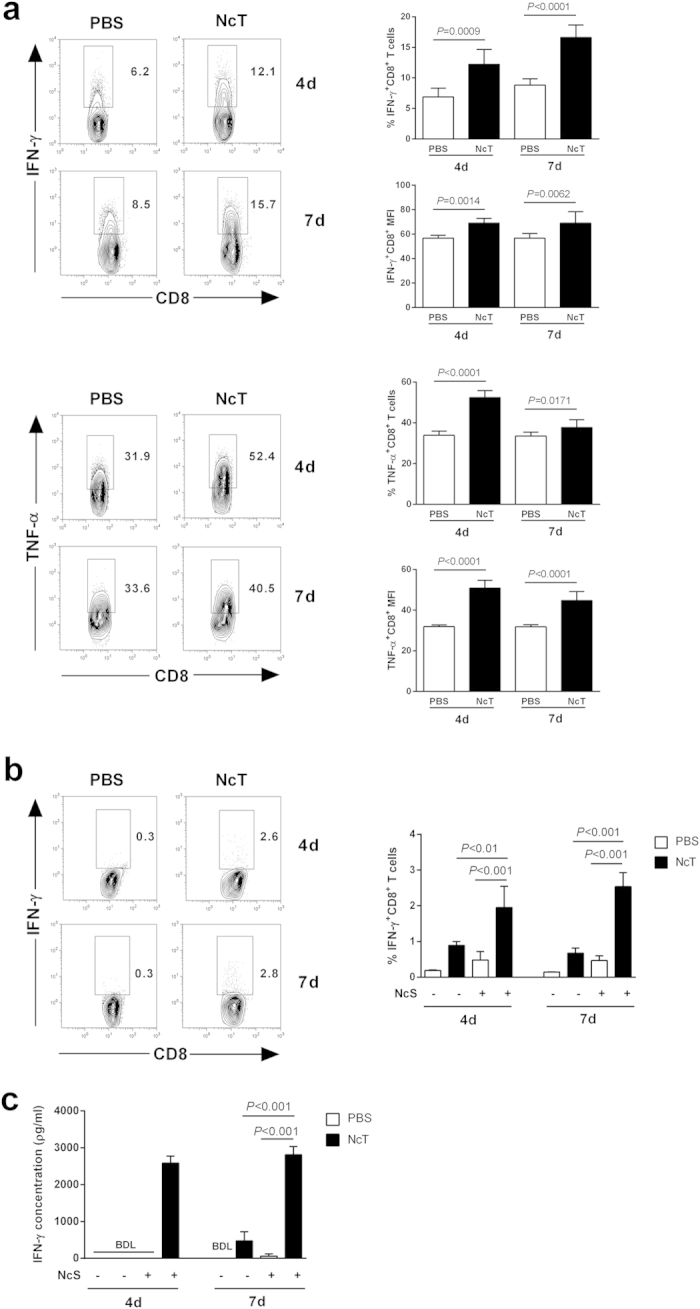
Increased production of INF-γ and TNF-α by CD8^+^ T cells of *N. caninum* infected mice. (**a**) Percentage of splenic CD8-gated T cells expressing IFN-γ or TNF-α of infected mice (NcT) and controls (PBS), detected by intracellular staining after stimulation with PMA/ionomycin. Mean fluorescence intensities due to respective cytokine staining are also presented. Bars represent means plus one SD of pooled data from two independent experiments (n = 6 for controls and 4-day infected mice and n = 9 for 7-day infected mice). Unpaired two-tailed *t*-test was used to compare parasite-inoculated vs respective control mouse groups. Statistical significance between infected mice and controls is indicated above bars. (**b**) Percentage of IFN-γ^+^ cells on total CD8^+^ T cells of infected mice (NcT) and controls (PBS) detected in *in vitro* splenocytes cultures non-stimulated (−) or stimulated for 16 h with *N. caninum* sonicates (+); n = 5 and n = 7 for non-stimulated and stimulated groups, respectively. Contour plots correspond to a representative example of CD8-gated T cells of the analysed samples. Analysis regions were set according to isotype control-stained samples. Numbers within contour plots correspond to the percentage of cells in the region shown. (**c**) IFN-γ concentration in the supernatants of splenocyte cultures non-stimulated (−) or stimulated for 16 h with *N. caninum* sonicates (+); n = 5 and n = 7 for non-stimulated and stimulated groups, respectively; BDL-below detection limit (15 pg/ml). Statistical significances between indicated pair groups on panels (**b**) and (**c**) were determined by one-way ANOVA and Tukey’s *post-hoc* test and are shown above bars.

IFN-γ plays a key role in the protective immune response to *N. caninum* infection as previously reported by others^24^. Therefore, production of this cytokine by CD8^+^ T cells was assessed in infected B6 mice and controls. As shown in Fig. 5a, an increased frequency of splenic CD8^+^IFN-γ^+^ T cells was found in the infected mice. Moreover, the mean fluorescence intensity due to IFN-γ staining was higher in CD8^+^ T cells from the infected mice than in non-infected controls (Fig. 5a). As shown in Supplementary Fig. S3, infected ScSn mice similarly displayed higher splenic CD8^+^IFN-γ^+^ T cell proportions than non-infected controls. In the infected B6 mice, the percentage of CD4^+^ T cells producing IFN-γ was also found above that of controls (Supplementary Fig. S4a). Interestingly, the proportions of CD4^+^ T cells producing IFN-γ in the infected *CD8a*^*−*/*−*^ mice did not differ from the ones found in the infected WT counterparts (Supplementary Fig. S5 and S4a, respectively).

**Figure 6:**
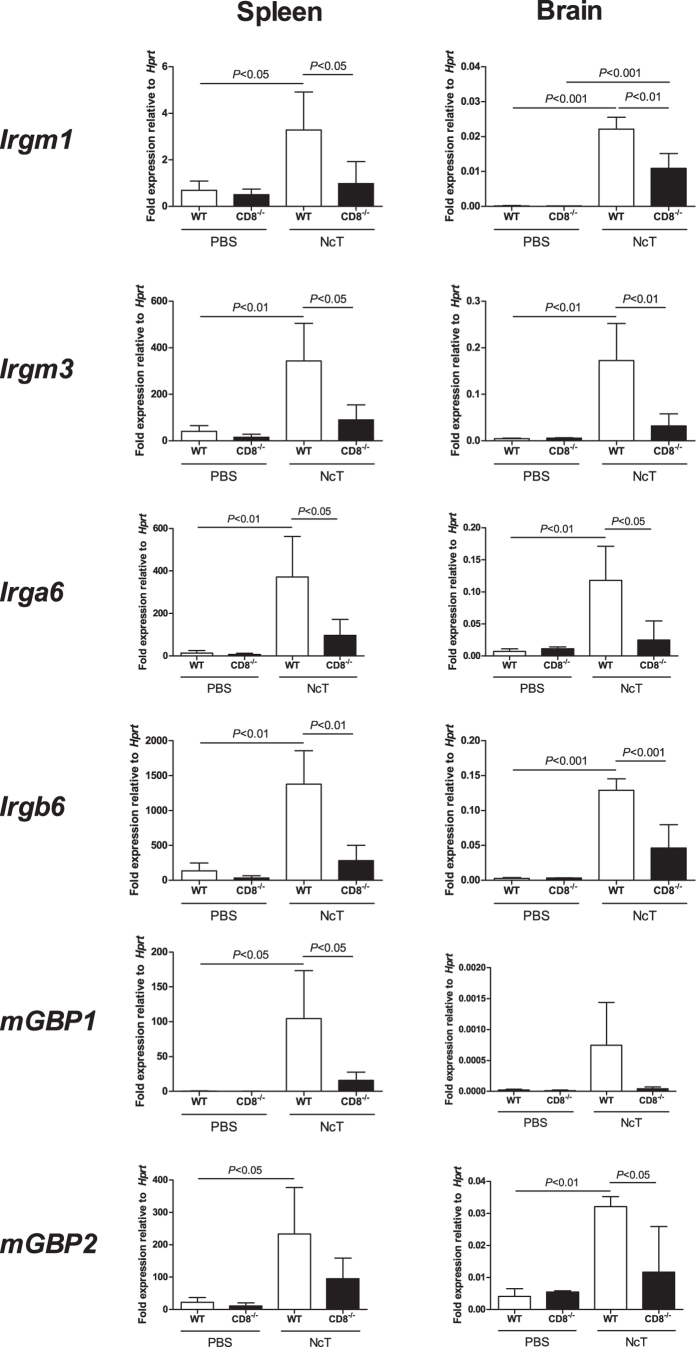
Lack of CD8^+^ T cells decreases IRG mRNA expression in infected mice. Relative levels of *Irgm1, Irgm3, Irga6, Irgb6, mGBP1* and *mGBP2* mRNA, normalized to hypoxanthine guanine phosphoribosyl transferase (*Hprt*) mRNA, detected by real-time PCR in the spleen and brain of WT and *CD8a*^*−*/*−*^ mice, as indicated, 7 days after i.p. injection of 1 × 10^7^ *N. caninum* tachyzoites (NcT; n = 4) or PBS (PBS; n = 3). Bars represent mean values of the respective group plus one SD. Statistical significance between infected mice and controls is indicated above bars (one-way ANOVA and Tukey’s *post-hoc* test).

**Figure 7:**
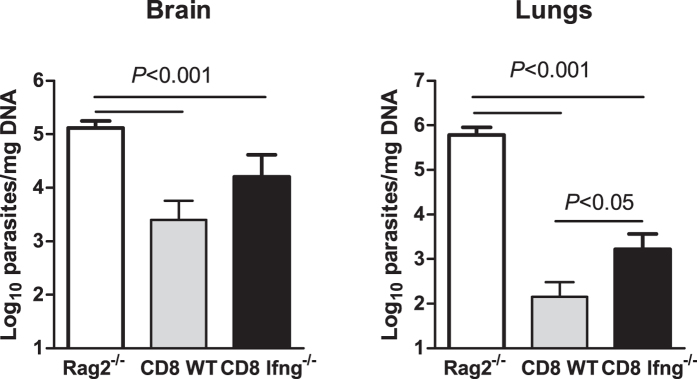
Protective effect of adoptively transferred *Ifng*^+/+^ CD8^+^ T cells in *N. caninum*-infected *Rag2*^*−*/*−*^ mice. Parasitic load in brain and lung tissue assessed by qPCR 7 days after i.p. infection with 1 × 10^7^ NcT of *Rag2*^*−*/*−*^ mice or *Rag2*^*−*/*−*^ mice reconstituted with WT CD4^+^ T cells and CD8^+^ T cells sorted from WT or *Ifng*^*−*/*−*^ mice, as indicated. Bars represent means plus one SD. Statistical significance between the different mouse groups (one-way ANOVA and Tukey’s *post-hoc* test) is indicated above bars (n = 4 for non-reconstituted *Rag2*^*−*/*−*^ mice and n=  10 per reconstituted group).

Higher proportions of IFN-γ-expressing CD8^+^ T cells, as well as of CD4^+^ T cells, were also detected in infected mouse spleen cell cultures stimulated with parasite antigens than in similarly stimulated cultures of control mouse splenocytes (Fig. 5b). Accordingly, higher IFN-γ levels were found in the supernatants of the antigen-stimulated cultures (Fig. 5c). Having determined that *N. caninum* infected mice present higher numbers and frequencies of IFN-γ^+^CD8^+^ T cells, we next evaluated the expression of immunity-related GTPases (IRG) Irgm1, Irgm3, Irga6, Irgb6, mGBP1 and mGBP2 in 7-day infected WT and *CD8a*^*−*/*−*^ mice, as these proteins were shown to be important immune effectors in mice infected with the related protozoan *T. gondii*^25^,^26^. As shown in Fig. 6, both infected mouse groups presented increased mRNA levels of the assessed IRG in the spleen and brain 7 days upon infection. However, these levels were significantly lower in infected *CD8a*^*−*/*−*^ mice than in infected WT controls. These results altogether show that CD8^+^ T cells contribute to this IFN-γ-dependent immune mechanism in the course of acute *N. caninum* infection. Other effector functions that might be activated by IFN-γ include those mediated by NADPH-dependent phagocyte oxidase or inducible nitric oxide synthase (NOS2)^27^. However, no significantly different parasitic loads were observed between 30-day infected WT, *p47phox*^*−*/*−*^ or *Nos2*^*−*/*−*^ mice (Supplementary Fig. S6). Also, expression of *Nos2* mRNA was not significantly different among 7-day infected mice and non-infected controls (Supplementary Fig. S7). These results indicate that production of NO and reactive oxygen species are not determinant host protective mechanisms in neosporosis. Taking these observations altogether into account, we next evaluated whether IFN-γ could be mediating the host protective effect of CD8^+^ T cells in the course of acute neosporosis. To this purpose, *Rag2*^*−*/*−*^ mice on a B6 background were reconstituted with CD4^+^ T cells sorted from *CD8a*^*−*/*−*^ mice and either CD8^+^ T cells sorted from IFN-γ-deficient (*Ifng*^*−/−*^) or WT donors. Both CD4^+^ and CD8^+^ T cells spontaneously proliferate and generate effector cells when adoptively transferred into lymphopenic RAG-deficient mice^28^. The success of the reconstitution was confirmed in each individual mouse by flow cytometric analysis of peripheral blood lymphocytes. By day 28 upon the cell transfer, the recipient mice were infected i.p. with 1 × 10^7^ NcT and lung and brain parasitic burdens were assessed by qPCR 7 days after the parasitic challenge. Non-reconstituted *Rag2*^*−*/*−*^ mice were similarly infected and analysed. As shown in Fig. 7, mice that received IFN-γ-expressing CD8^+^ T cells presented a significantly lower parasitic burden in the lungs than those that received IFN-γ-deficient CD8^+^ T cells. A slightly lower parasitic burden was also observed in the brain, but did not reach statistical significance. The mouse group reconstituted with WT donor CD8^+^ T cells presented higher numbers of splenic CD8^+^ T cells (Supplementary Table S1). However, no correlation was found between the total number or percentage of splenic CD8^+^ T cells and the detected parasitic burden in the brain (r^2 ^= 0,02261 and r^2^ = 0,002939, respectively) or lungs (r^2 ^= 0,04955 and 0,03606, respectively). Non-reconstituted *Rag2*^*−*/*−*^ mice presented significantly higher parasitic burdens in brain and lung tissue than any reconstituted group (Fig. 7). Lack of Rag2 expression makes the mice lethally susceptible to this parasite (Supplementary Fig. S8).These results altogether show that IFN-γ produced by CD8^+^ T cells mediates their host protective effect against neosporosis. Because an increased proportion of CD8^+^ T cells producing TNF-α was also detected in the spleen of *N. caninum* infected mice (Fig. 5a), we tested the possible contribution of TNF-α to protection using TNF-α-deficient (*Tnf*^*−/−*^) mice. Infected *Tnf*^*−/−*^ mice and WT controls presented similar parasitic burdens in the brain (4.39 ± 0.51 vs 4.84 ± 0.61 log_10_ parasites/mg DNA, respectively; *P *= 0.3027, n = 4) and lungs (3.33 ± 0.88 vs 3.23 ± 2.18 log_10_ parasites/mg DNA; *P *= 0.9348, n = 4), 7 days upon i.p. infection. In the infected WT mice, no significant difference was found in the percentage of splenic TNF-α^+^CD4^+^ as compared to sham-infected controls (Fig. S4). These results indicate that TNF-α plays a minor role in protection against acute neosporosis.

## Discussion

CD8^+^ T cells can work as CTL or as cytokine secreting cells. These cells have been extensively studied in the context of protozoan infections^15^ including those caused by the *N. caninum* closely related pathogen *Toxoplasma gondii*^13^,^29^,^30^,^31^. However, the role of CD8^+^ T cells in the course of neosporosis has only been addressed in a few studies^9^,^10^,^14^,^32^,^33^. Here, we show that mice lacking CD8^+^ T cells are more susceptible to *N. caninum* infection than their WT counterparts during the acute phase of infection. This higher susceptibility was also evident at a later time in 40-day infected mice. This result is in agreement with a previous study in which a mild effect in protecting mice against *N. caninum* infection was suggested for CD8^+^ T cells as assessed by using a CD8 T cell-depleting mAb^9^. Moreover, as we show here, adoptive transfer of CD8^+^ lymphocytes obtained from infected *N. caninum*-resistant ScSn mice into lethally susceptible ScCr recipients, prolonged their survival but did not confer complete protection from infection. The lack of complete protection observed in the ScCr mice receiving CD8^+^ T cells may reflect the need of IL-12-dependent CD4^+^ T cell or NK cell activation, previously shown to be important in mice infected with the related parasite *T. gondii*^34^,^35^. A previous study reported that adoptive transfer of *in vivo N. caninum*-primed CD8^+^ T cells prior to infection precipitated neurological disease in resistant BALB/c mice challenged with NcT^14^. The immunocompetent status of these recipients might have contributed to the reported effect, a likely consequence of immunopathology. Our results altogether indicate that CD8^+^ T cells have a host protective role in this infection. In accordance Ritter *et al.*^36^ have shown that β2 microglobulin (β2M)-deficient mice, which also lack CD8^+^ T cells, are lethally susceptible to neosporosis. The higher susceptibility to *N. caninum* infection of β2M-deficient mice as compared to the one we observed in *CD8a*^*−*/*−*^ mice, suggests that mechanisms other than those dependent on CD8^+^ T cells may also be involved in the control of neosporosis as the immune deficit of β2M-deficient mice goes beyond the lack of CD8^+^ lymphocytes^37^,^38^. The lower parasitic burden detected in the brain of *CD8a*^*−/−*^ mice 40 days post-infection as compared to that detected in 7-day infected animals also indicates that other cell populations than CD8^+^ T cells mediate immune protection in the brain. CD4^+^ T cells or NKT cells may be likely candidates as could be suggested by antibody-mediated depletion studies^9^,^39^.

The protective effect of CD8^+^ T cells was demonstrated in *T. gondii* infected mice in experiments also involving adoptive transfer^40^,^41^,^42^ or depletion^43^ of this lymphocyte population. Interestingly, previous *in vivo* observations showed that infection with *N. caninum* was able to protect against lethal *T. gondii* infection by the induction of CD8^+^ T cells immunoreactive to both parasites^33^. Nevertheless, CD8^+^ T cells appear to play a more prominent role in protecting the murine host to toxoplasmosis than to neosporosis as mice defective in CD8^+^ T cells succumb when challenged with *T. gondii*^44^. These findings suggest that despite the extensive similarities between these parasites, the host protective immune response may present different features in each case.

The surface CD44^+^CD62L^low^ phenotype was previously used to assess CD8^+^ T cell function and cytotoxic activity in *T. gondii* infected mice^42^ and the CD62^low^ phenotype was previously reported to be characteristic of a T CD8^+^ effector subpopulation in mice infected with this parasite^45^. Phenotypic characterization of the CD8^+^ T cells isolated from infected WT B6 and ScSn mice showed increased surface expression of the activation marker CD44 as well as a decrease in CD62L expression, as compared to sham-infected controls. Moreover, a higher frequency of granzyme B-expressing CD8^+^ T cells was found in the infected B6 WT mice, as compared to sham-infected controls. These surface and intracellular phenotypes were also found in CD8^+^ T cells of mice infected with other protozoan parasites and indicate T cell activation and CTL differentiation^46^,^47^,^48^. In accordance with this activated phenotype, increased numbers of IFN-γ^+^CD8^+^ T cells were also observed in infected immunosufficient mice. Noteworthy, CD4^+^ T cells, which have been previously shown to be important effectors in the immune response to *N. caninum*^9^,^32^ similarly displayed an activated phenotype and produced IFN-γ in the parasite challenged mice. As it has been previously demonstrated and also shown here, IFN-γ is a crucial cytokine for host resistance to *N. caninum*^24^,^49^. Given that infected *Rag2*^*−*/*−*^ mice adoptively transferred with *Ifng*^*−/−*^ CD8^+^ T cells presented higher parasitic burdens than counterparts transferred with *Ifng*^*+/+*^ CD8^+^ T cells, this implicates IFN-γ in the host protective role of this lymphocytic population against neosporosis. Mice reconstituted with *Ifng*^*−*/*−*^ CD8^+^ T cells presented lower parasitic burdens than non-reconstituted *Rag2*^*−/−*^ mice. IFN-γ produced by co-transferred WT CD4^+^ T cells and possible IFN-γ-independent CD8^+^ T cell mechanisms may have contributed to the observed protection. The specific effector mechanisms by which IFN-γ could mediate protection remain to be completely elucidated. Recently, up-regulated expression of IFN-γ-dependent IRG mRNA has been shown to occur in the brain of *N. caninum* infected mice^50^. Here we have also shown that mRNA levels of several IRG are up-regulated in the brain and spleen of infected WT and *CD8a*^*−*/*−*^ mice. However, mice lacking CD8^+^ T cells generally presented lower levels of IRG mRNA than WT counterparts upon infected with *N. caninum*. This indicates that these proteins, for which a significant role in resistance to *T. gondii* has been proved^25^, could also mediate the protective role of CD8-T cell-derived IFN-γ in neosporosis. In addition to activation of IRG, STAT1-dependent production of nitric oxide and reactive oxygen species may be plausible candidates, which have been proven important for *T. gondii* clearance^51^. However, we found no evidence for significantly increased transcription of *Nos2* in the infected mice. Moreover, we show here that *Nos2*^*−*/*−*^ and *p47Phox*^*−*/*−*^ mice survived infection without evident clinical signs and presented similar parasitic burdens to those of WT controls 30 days upon infection. A previous report that used *Nos2*-deficient mice of the BALB/c background has also shown that this enzyme does not play a major protective role against acute or chronic *N. caninum* infection^36^. All these results indicate that mechanisms involving either production of nitric oxide or reactive oxygen species do not seem to be crucial in containing acute neosporosis.

Higher frequencies and numbers of splenic CD8^+^ T cells producing TNF-α were also found in the infected mice. However, as TNF-α-deficient mice did not show an increased susceptibility to this parasite, it is unlikely that this cytokine plays a major role in the host protective effect mediated by CD8^+^ T cells in the acute phase of *N. caninum* infection. Indeed, previous studies provided *in vitro*^52^ and *in vivo*^36^ evidence for a less important, although non-negligible, role of TNF-α in host protection against *N. caninum* infection, as compared to that of IFN-γ. A predominant role of CD8^+^ T cell-produced IFN-γ over that of TNF-α was also found in protection against liver-stage *Plasmodium* infection, as previously reviewed^53^.

Previous studies suggested that perforin-dependent cytotoxicity mediated by antigen-specific CD4^+^ T cells differentiated *in vivo* or by *in vitro* activated NK cells could be a host protective mechanism in cattle infected with *N. caninum*^7^,^10^,^11^. Using CD107a (LAMP-1) surface expression as a surface marker indicative of T cell cytotoxic activity^23^, we found evidence supporting a cytotoxic function of CD8^+^ T cells in infected B6 mice. However, as WT and *Prf1*^*−*/*−*^ B6 mice infected with *N. caninum* presented similar parasitic burdens, perforin-dependent cytotoxicity does not appear to be a key mechanism involved in the parasite control during acute infection. As we observed that CD8^+^ as well as CD4^+^ T cells of *N. caninum* infected *Prf1*^*−*/*−*^ B6 mice responded by producing IFN-γ to the same extent as infected WT counterparts (Supplementary Fig. S9), this could account for the lack of increased susceptibility. Accordingly, CTL activity was previously shown to be non-essential^54^ albeit non-negligible^29^ in the immune response to acute *T. gondii* infection. Therefore, the protective effect of CD8^+^ lymphocytes in *N. caninum* infection seems to rely more on the production of IFN-γ than on cytotoxicity. Similarly, prevention of toxoplasmic encephalitis in BALB/c mice was found to depend on IFN-γ production rather than on perforin-mediated cytotoxicity^55^.

The CD8^+^ T cell population has been shown to be host protective in infections caused by apicomplexan protozoa^15^. The results presented here directly show that CD8^+^ T cells also have a host protective effect in murine *N. caninum* infection and implicate IFN-γ production as a major effector mechanism. Previous reports have shown that stimulation by immunization of parasite antigen-specific IFN-γ-producing CD8^+^ T cells significantly reduced parasitic burden in mice infected with *T. gondii*^56^,^57^. Our results provide evidence suggesting that stimulation of these lymphocyte cells by means of immunization could also be worth exploring towards immune prevention of neosporosis.

## Methods

### Mice

Female B6 WT mice were obtained from Charles River (Barcelona, Spain), and female *Cd8a*^*−*/*−*^ and *Prf1*^*−*/*−*^ mice on B6 background were obtained from Jackson Laboratories (Bar Harbor, ME, USA). Female ScCr and ScSn mice were obtained from the Gulbenkian Institute of Science (Oeiras, Portugal). ScCr mice are homozygous for a deletion encompassing *Tlr4* gene and harbour a point mutation that results in the precocious termination of the transcript for the IL-12Rβ2 chain in the IL-12 receptor^21^. The ScSn mice are TLR4-competent and have no defective IL-12-mediated responses^58^. These mice were bred at the animal facilities of Instituto Abel Salazar during the experiments. Female *Ifng*^*−/−*^ and *Rag2*^*−*/*−*^ B6 mice were obtained from Jackson Laboratories and *Tnf*^*−/−*^ B6 mice were purchased from B&K Universal (Hull, UK). Female *p47phox*^−/−^ B6 mice were purchased from Taconic (Lille Skensved, Denmark). iNOS-deficient C57BL/6 mice (*Nos2*^*−*/*−*^)^59^ were bred in our facilities after backcrossing the original strain (kindly provided by Drs J. Mudgett, J. D. MacMicking and C. Nathan, Cornell University, New York, NY, USA) onto a B6 background for seven generations. All these mice were housed and bred at Instituto de Biologia Celular e Molecular (IBMC) animal facilities. Female B6 WT mice in the experiments using *Rag2*^*−/−*^ and *Ifng*^*−/−*^ animals were bred at IBMC. Hiding and nesting materials were provided as enrichment. Procedures involving mice were performed according to the European Convention for the Protection of Vertebrate Animals used for Experimental and Other Scientific Purposes (ETS 123) and 86/609/EEC Directive and Portuguese rules (DL 129/92). All experimental protocols were approved by the competent national board (Direcção Geral de Veterinária, documents 0420/000/000/2007, 0420/000/000/2008, 0420/000/000/2010).

### Parasites

*N. caninum* tachyzoites (NcT) (Nc-1, ATCC® 50843) were propagated by serial passages in VERO cell cultures, maintained in Minimal Essential Medium (MEM) containing Earle’s salts (Sigma, St. Louis, MO, USA), supplemented with 10% fetal calf serum (PAA laboratories, Pasching, Austria), L-Glutamine (2 mM), Penicillin (200 IU/ml) and Streptomycin (200 g/ml) (all from Sigma), in a humidified atmosphere with 5% CO_2_ at 37 °C. Free parasitic forms of *N. caninum* were obtained as previously described^4^. Briefly, infected VERO cells were cultured until the host cell monolayer was 70% destroyed. Free parasites and adherent cells were recovered using a cell scraper and centrifuged at 1,500 × g for 15 min. The pellet was passed through a 25 G needle and then washed three times in PBS by centrifugation at 1,500 × g for 15 min. The resulting pellet was resuspended and passed through a PD-10 desalting column, containing Sephadex™ G-25M (GE Healthcare, Freiburg, Germany). Tachyzoites concentration was determined in a haemocytometer.

### Challenge infections and collection of biological samples

*N. caninum* infections in B6 WT, *Cd8a*^*−*/*−*^, *Prf1*^*−*/*−*^, *Rag2*^*−*/*−*^, *Tnf*^*−*/*− −*/*−*^, *p47phox*^−/−^, *Nos2*^*−*/*−*^ or in ScSn mice were performed by i.p. inoculation of 1 × 10^7^ freshly isolated NcT in 500 μL of PBS. Sham-infected controls were similarly injected with PBS alone. In the euthanized WT B6 and ScSn mice, spleens were aseptically removed 4 and/or 7 days after infection, for the analysis of the immune response and *in vitro* cell cultures. The lungs and brain were collected 7 days after infection in B6 WT, *Cd8a*^*−*/*−*^, *Prf1*^*−*/*−*^, *Rag2*^*−*/*−*^, and *Tnf*^*−−*/*−*^ mice. Brains were also collected 30 days after infection in B6 WT, *Nos2*^*−*/*−*^, and *p47phox*^*−*/*−*^ mice or 40 days after infection in B6 WT and *Cd8a*^*−*/*−*^ mice, and stored at −20 °C for DNA extraction. Infection of ScCr mice was made by i.p. inoculation of 5 × 10^5^ freshly isolated NcT in 500 μL of PBS, 16h after the adoptive transfer of CD8^+^ T cells. These mice were monitored twice a day for morbidity signs and the following humane end-points were used to determine the end of the experiment: 15% weight loss, paralysis of the posterior limbs, severe dehydration or decrease in body temperature.

### Flow cytometry

Spleens were aseptically removed, homogenised in HBSS (Sigma) and, when necessary, red blood cells were lysed. The following mAb were used (at previously determined optimal dilutions) for surface antigen staining after pre-incubation with anti-mouse CD16/CD32 for FcγR blocking: anti-mouse CD3 Phycoerythrin (PE)- or PE-Cy5-conjugate (clone 145-2C11), anti-mouse CD4 Fluorescein isothiocyanate (FITC)- or peridinin-chlorophyll protein-cychrome (PerCP-Cy5.5)-conjugate (clone RM4–5), anti-mouse CD8 FITC- or PerCP-Cy5.5-conjugate (clone 53–6.7), anti-mouse CD44 PE-cychrome 7 (PE-Cy7)-conjugate (clone IM7), anti-mouse CD62L PE-conjugate (clone MEL-14) (all from BD Biosciences, San Jose, CA, USA) and CD107a (Lamp-1) PE-conjugate (clone ebio1D4B) (eBioscience, San Diego, CA, USA). For intracellular cytokine detection, cells were counted and plated in round bottom 96 plates (Nunc, Roskilde, Denmark), at a concentration of 5 × 10^6^ cells/ml in RMPI-1640 (Sigma) supplemented with 10% fetal calf serum (PAA laboratories), HEPES (10 mM), Penicillin (200 IU/ml) and Streptomycin (200 g/ml) (all from Sigma), β-mercaptoethanol (0.1 mM) (Merk, Darmstadt, Germany) (complete RMPI). Cells were incubated in a humidified atmosphere with 5% CO_2_ at 37 °C for 5 h under stimulation with phorbol myristate acetate (PMA; Sigma), 10 ng/ml, and ionomycin (Merk), 1 μg/ml in the presence of 10 μg/ml Brefeldin A (Sigma), or similarly incubated for 16 h under stimulation with 100 μg/ml *N. caninum* sonicates, and 10 μg/ml Brefeldin A was added for the last 5 h. Upon incubation with the different stimuli, cells were recovered, and incubated with anti-mouse CD16/CD32, prior to staining with anti-CD3 and anti-CD8 mAbs. Following extracellular staining the cells were washed, fixed, and permeabilized with 0.05% saponin (Sigma) PBS solution and intracytoplasmic staining was carried out with anti-IFN-γ FITC-conjugate (clone XMG1.2; BD Biosciences), anti-TNF-α PE-Cy7-conjugate (clone MP6-XT22; BioLegend, San Diego, CA) mAb. For granzyme B detection, cells were incubated for 4 h in complete RPMI with 10 μg/ml Brefeldin A, without PMA/ionomycin stimulus. Intracellular granzyme B staining was performed as described above for cytokine detection by using specific anti-mouse FITC-conjugate mAb (clone NGZB; eBioscience). Antibody-labelled cells were analyzed in an EPICS XL flow cytometer using the EXPO32ADC software (Beckman Coulter, Miami, FL, USA) or in a FACSCalibur^TM^ using the CellQuest software (Becton Dickinson, San Jose, CA, USA). A minimum of 150,000 events were acquired per sample. The collected data files were analysed in FlowJo version 9.7.5. (Tree Star inc., Ashland, OR, USA).

### T cell sorting and adoptive transfer

For the reconstitution of T cell populations in *Rag2*^*−*/*−*^ mice, CD4^+^ T cells were isolated from pooled spleens of *Cd8a*^*−*/*−*^ mice by using negative magnetic cell sorting with a CD4^+^ T-cell isolation kit (Miltenyi Biotech, Inc., Auburn, CA, USA). CD8^+^ T cells were isolated from pooled spleens of WT or *Ifng*^*−*/*−*^ mice by using negative magnetic cell sorting with a CD8^+^ T-cell isolation kit (Miltenyi Biotech) and were further purified by flow cytometry cell sorting in a FACSAria equipped with the FACSDiva software (Becton Dickinson) upon staining with anti-CD8 mAb FITC-conjugate. Purity of CD8^+^ sorted cells was higher than 99.0%. Purity of magnetic sorted CD4^+^ T cells was assessed in an EPICS XL flow cytometer using the EXPO32ADC software (Beckman Coulter) after staining with anti-CD3 PE-conjugate and anti-CD4 PerCP-Cy5.5-conjugate and ranged between 90–95%. *Rag2*^*−/−*^ were divided in two groups and were injected intravenously with 1.5 × 10^6^ purified CD4^+^ T cells, and with 1.5 × 10^6^ purified CD8^+^ T cells of either WT (n = 10) or *Ifng*^*−/−*^ (n = 10) mice. Infection of both mouse groups was performed 28 days after T cell administration, when mice already showed CD4^+^ and CD8^+^ T cell reconstitution, as assessed by flow cytometry in blood samples collected from the submandibular vein.

To obtain purified CD8^+^ T cells, spleens of ScSn mice infected i.p. with 500 μl of PBS containing 1 × 10^7^ NcT or sham-infected with PBS alone were removed and homogenized in Hanks balanced salt solution (HBSS, Sigma) and red blood cells were lysed. Cells were incubated with anti-mouse CD8 FITC-conjugate mAb. Flow cytometry cell sorting was performed as described above. The purity of the separated cells was >98%. Next, 1 × 10^6^ CD8^+^ T cells purified from infected or control mice were respectively adoptively transferred into naïve ScCr mice by tail vein injection.

### DNA extraction

DNA from the brain and lungs was extracted by using previous described methodology^49^. Briefly, brains and lungs were digested overnight at 55 °C in a 1% sodium dodecyl sulphate solution containing 1 mg/ml Proteinase K (USB Corporation, Cleveland, OH, USA). DNA was then extracted by the phenol (Sigma)-chloroform (Merck) method followed by ammonium acetate/ethanol precipitation.

### PCR for the detection of NcT

The parasite burden in the brain and lungs of infected mice was assessed as previously described^60^ by a quantitative real-time PCR (qPCR) analysis of the parasite DNA performed in a Corbett rotor gene 6000 system (Corbett life science, Sydney, Australia). Product amplification was performed with 500–1000 ng of template DNA using KAPPA Probe fast universal qPCR kit (Kappa biosystems, Wilmington, MA, USA) for the amplification of a 103 bp sequence of the Nc5 region of *N. caninum* genome using the primers NcA 5′ GCTACCAACTCCCTCGGTT 3′ and NcS 5′ GTTGCTCTGCTGACGTGTCG 3′ both at a final concentration of 0.2 μM and the florescent probe FAM-CCCGTTCACACACTATAGTCACAAACAAAA-BBQ (all designed and obtained from TIB-Molbiol, Berlin, Germany). The DNA samples were amplified using the following program: 95 °C for 3 min, 95 °C for 5 sec, 60 °C for 20 sec with fluorescence acquisition, the second and third step were repeated 45 times. Length of the amplified DNA was confirmed in a 3% agarose gel stained with ethidium bromide. In all runs parasite burden was determined by interpolation of a standard curve performed with DNA isolated from *N. caninum* tachyzoites, ranging from 2 to 2 × 10^5^ parasites, included in each run. Data were analyzed in the Rotor gene 6000 software v1.7 (Corbett life science) and expressed as log_10_ parasites per mg of total DNA.

### RNA isolation and real-time PCR analysis

Total RNA was extracted from whole brain tissue samples or from 5 × 10^6^ splenocytes 7 days after infection, using TriReagent™ (Sigma-Aldrich) according to manufacturer’s instructions. All RNA samples were recovered in 10 μL of nuclease-free H_2_O and quantified using Nanodrop ND-1000 apparatus (Thermo Scientific). For *Irga6* transcript quantitation, RNA samples were treated with DNase I (Invitrogen) prior to synthesis of cDNA, according to manufacturer’s instructions. Synthesis of cDNA was performed from 1–2.5 μg of total RNA prepared as described above in a 10 μl final volume using Maxima® First Strand cDNA Synthesis kit for RT-qPCR (Fermentas, Thermo Scientific), according to manufacturer’s instructions. The PCR program run (25 °C, 10 min; 50 °C, 30 min; 85 °C, 5 min) was performed in a TProfessional Basic Thermocycler (Biometra GmbH, Goettingen, Germany). Real-time PCR was then used for the semi-quantification of *Nos2*, *Irgm1, Irgm3, Irga6, Irgb6, mGBP1* and *mGBP2* mRNA expression levels with the Kapa SYBR Fast qPCR Kit (Kapa Biosystems Inc) in a Rotor-Gene 6000 (Corbett life science), following previously described methodologies, with slight modifications^60^,^61^,^62^,^63^. As reference gene we used hypoxanthine guanine phosphoribosyl transferase (*Hprt*). For the quantification of mRNA expression levels, the reaction was performed in a final volume of 10 μL containing 0.2 μM of each specific primer: *Hprt* forward: ACA TTG TGG CCC TCT GTG TG, *Hprt* reverse: TTA TGT CCC CCG TTG ACT GA, *Nos2* forward: CCA AGC CCT CAC CTA CTT CC; *Nos2* reverse: CTC TGA GGG CTG ACA CAA GG; *lrgm1* forward: CTC TGG ATC AGG GTT TGA GGA GTA, *lrgm1* reverse: GGA ACT GTG TGA TGG TTT CAT GAT A; *Irgm3*forward: CTG AGC CTG GAT TGC AGC TT, *Irgm3* reverse: GTC TAT GTC TGT GGG CCT GA; *Irga6* forward: CTT GGA CAG TGA TTT GGA GAC, *Irga6* reverse: AGT ACC CAT TAG CCA AAC AG; *Irgb6* forward: TTG CCA CCA GAT CAA GG TCA C, *Irgb6* reverse: CAA GGT GAT GTC ATA TTC AGA GAT G; *mGBP1*forward: CAG ACT CCT GGA AAG GGA CTC, *mGBP1* reverse: CTT GGA TTC AAA GTA TTT TCT CAG C; *mGBP2* forward: TGA GTA CCT GGA ACA TTC ACT GAC, *mGBP2* reverse: AGT CGC GGC TCA TTA AAG C (all from Tib Molbiol) and 1× Master Mix plus 1 μL of the newly-synthesized cDNA diluted 1/10. The PCR program run was as follows: 1) denaturation at 95 °C, 5 min 2) amplification in 35 cycles (95 °C, 10 s; 62 °C, 20 s). We analyzed real-time PCR data by the comparative threshold cycle (CT) method. Individual relative gene expression values were calculated using the following formula: 2 ^− (CT gene of interest − CT constitutive gene)^^64^.

### Statistical analysis

Statistical analyses were performed using GraphPad software (Version 6.0, GraphPad Software Inc, La Jolla, CA, USA). Unless otherwise indicated, statistical analysis between parasite-inoculated mice vs respective control groups was performed using unpaired two tailed student’s *t*-test. Column graphs are represented showing means plus one SD.

## Additional Information

**How to cite this article**: Correia, A. *et al.* Predominant role of interferon-γ in the host protective effect of CD8^+^ T cells against *Neospora caninum* infection. *Sci. Rep.* **5**, 14913; doi: 10.1038/srep14913 (2015).

## Supplementary Material

Supplementary Information
