# Supplementary material for: Predominant role of interferon-γ in the host protective effect of CD8+ T cells against Neospora caninum infection
Source: Sci Rep. 2015 Oct 9;5:14913. doi: 10.1038/srep14913 (PMC4598874; doi:10.1038/srep14913)
Supplement: Supplementary Information [file srep14913-s1.pdf]

Predominant role of interferon-γ in the host protective effect of $CD8^+$ T cells against *Neospora caninum* infection

Alexandra Correia[a], Pedro Ferreirinha[a,b], Sofia Botelho[b], Ana Belinha[b], Catarina Leitão[a], Íris Caramalho[c], Luzia Teixeira[b,d], África González-Fernandéz[e], Rui Appelberg[a,b], Manuel Vilanova[a,b*]

Instituto de Investigação e Inovação em Saúde, Universidade do Porto, and IBMC - Instituto de Biologia Molecular e Celular, Universidade do Porto, 4150-180 Porto, Portugal[a]; ICBAS - Instituto de Ciências Biomédicas de Abel Salazar, Universidade do Porto, 4050-313 Porto, Portugal[b]; Instituto de Medicina Molecular, Faculdade de Medicina, Universidade de Lisboa, 1649-028 Lisboa, Portugal[c]; UMIB-Unidade Multidisciplinar de Investigação Biomédica, Universidade do Porto, Porto[d] Inmunología, Centro de Investigaciones Biomédicas (CINBIO), Instituto de Investigación Biomédica, Universidade de Vigo, Campus Lagoas Marcosende, E-36200 Vigo, Spain[e].

* vilanova@icbas.up.pt

Supplementary Figure S1: CD4$^+$ T cells are activated and expand in the spleen of *N. caninum*-infected C57BL/6 WT mice

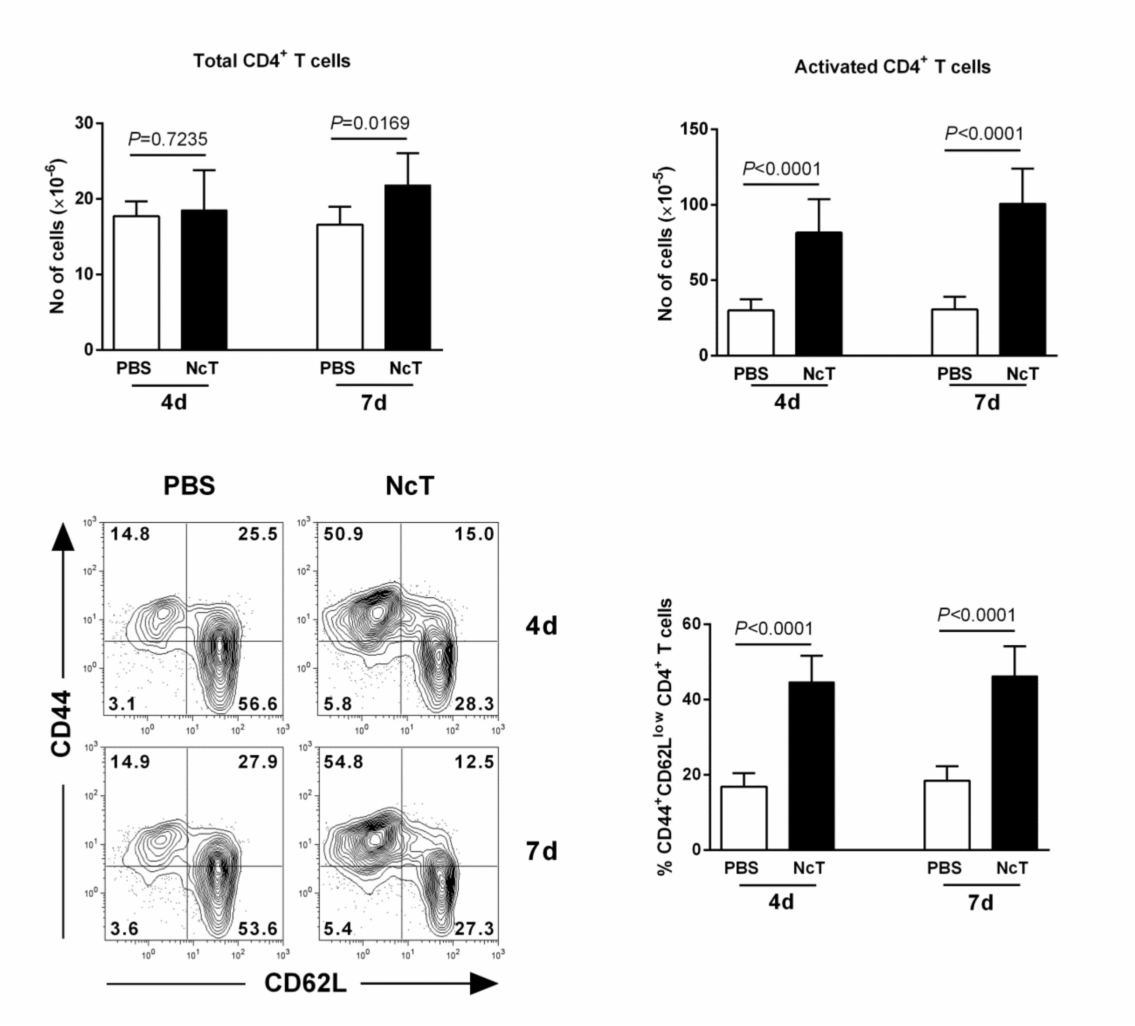

Numbers of total and activated (CD44$^+$CD62L^{low}) CD4$^+$ T cells, and percentage of activated CD4$^+$ T cells, as indicated, detected in the spleen of C57BL/6 mice infected i.p. with 1×10^7 *N. caninum* tachyzoites (NcT), or sham-infected injected i.p. with PBS alone (PBS), 4 and 7 days upon the parasitic challenge.. Bars represent means plus one SD of pooled data from two independent experiments (n=6 and n=9 for control and infected groups, respectively). Unpaired two-tailed t-test was used to compare parasite-inoculated vs respective control mouse groups. Statistical significance between infected mice and controls is indicated above bars. Contour plots correspond to a representative example of CD4-gated T cells of the analysed samples. Quadrants and regions were set according to isotype control-stained samples. Numbers within contour plots correspond to the percentage of cells in each quadrant.

Supplementary Figure S2: CD8-deficient mice are more susceptible to long-term *N. caninum* infection than control WT mice

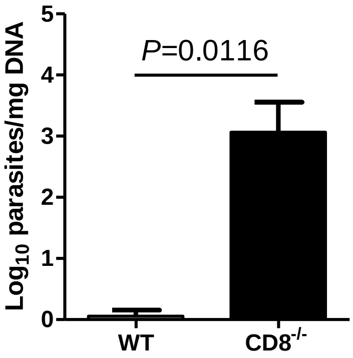

Parasitic load of brain tissue assessed by qPCR specific for *N. caninum* DNA in WT or *CD8a*$^{-/-}$ mice, as indicated, 40 days after i.p. inoculation of 1×10^7 NcT. Bars represent means plus one SD of pooled data from two independent experiments (n=6 per group). Statistical significance between infected mice and controls is indicated above bars.

Supplementary Figure S3: CD8$^+$ T cells are activated and produce IFN-γ in *N. caninum*-infected C57BL/10 ScSn mice

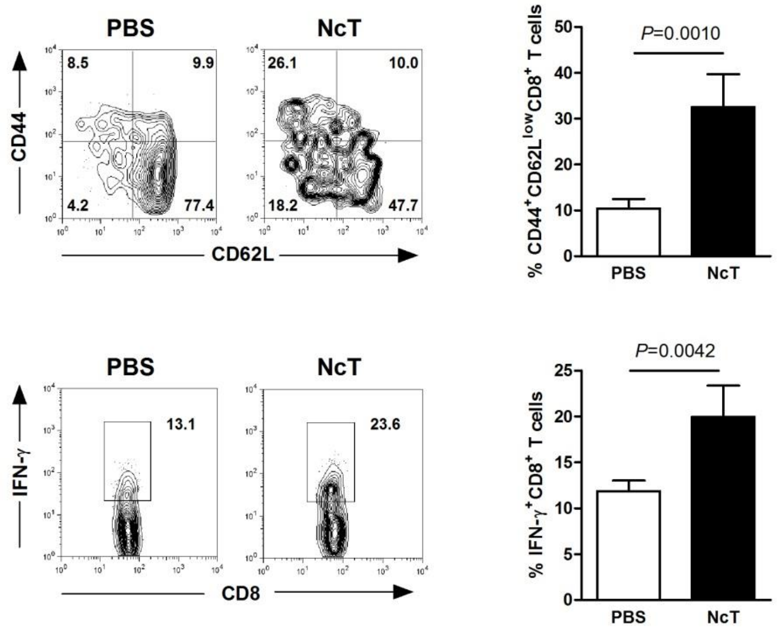

Proportions of activated (CD44$^+$CD62L^{low}) and of IFN-γ$^+$ CD8$^+$ T cells, as indicated, detected in the spleen of ScSn mice 7 days upon infection i.p. with 1×10^7 NcT (NcT) or sham-infection with PBS alone (PBS). Bars represent means plus one SD of four mice per group. Unpaired two-tailed t-test was used to compare parasite-inoculated vs respective control mouse groups. Statistical significance between infected mice and controls is indicated above bars. Contour plots correspond to a representative example of CD8-gated T cells of the analysed samples. Quadrants and regions inside contour plots were set according to isotype control-stained samples. Numbers within contour plots correspond to the percentage of cells in each quadrant or region.

Supplementary Figure S4: IFN-γ and TNF-α expression in *in vitro* stimulated splenic CD4^{+} T cells of *N. caninum* infected mice and controls

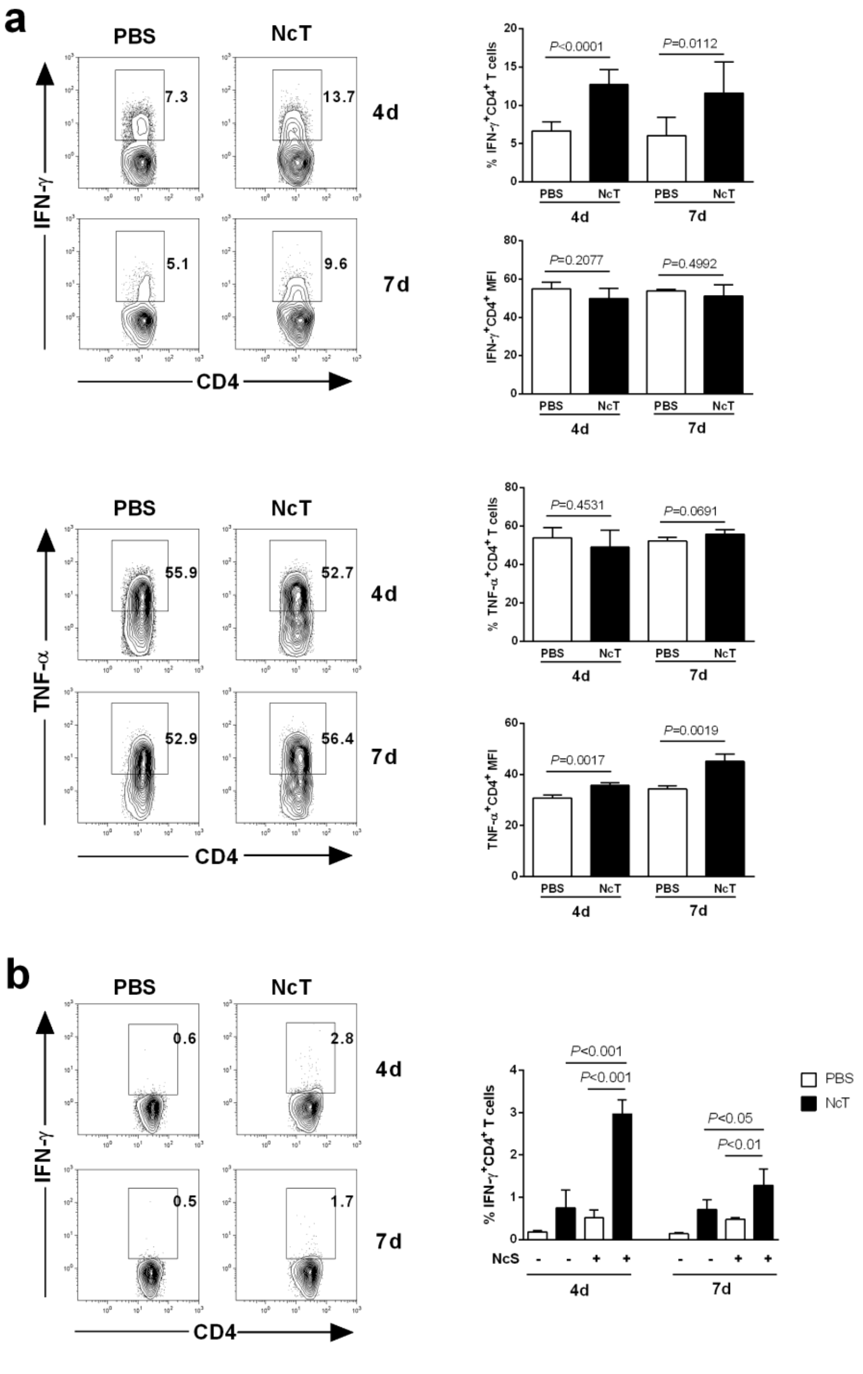

(a) Percentage of splenic CD4-gated T cells expressing IFN-γ or TNF-α of $1{\times}10^7$ NcT-infected mice (NcT) and sham-infected controls (PBS), detected by intracellular staining after stimulation with PMA/ionomycin. Mean fluorescence intensities (MFI) due to respective cytokine staining are also presented. Bars represent means plus one SD of pooled data from two independent

experiments (n=6 for controls and n=9 for infected mice). Statistical significance between infected mice and controls is indicated above bars. (b) Percentage of IFN-γ^{+} cells on total CD4^{+} T cells of infected mice (NcT) and controls (PBS) detected in *in vitro* splenocytes cultures non stimulated (-) or stimulated for 16 h with *N. caninum* sonicates (+); n=5 and n=7 for non-stimulated and stimulated groups, respectively. Contour plots correspond to a representative example of stimulated CD4-gated T cells of the analysed samples. Analysis regions were set according to isotype control-stained samples. Numbers within contour plots correspond to the percentage of cells in the region shown.

Supplementary Figure S5: Proportions of IFN-γ-expressing CD4^{+} T cells in *CD8a*$^{-/-}$ mice infected with *N. caninum*.

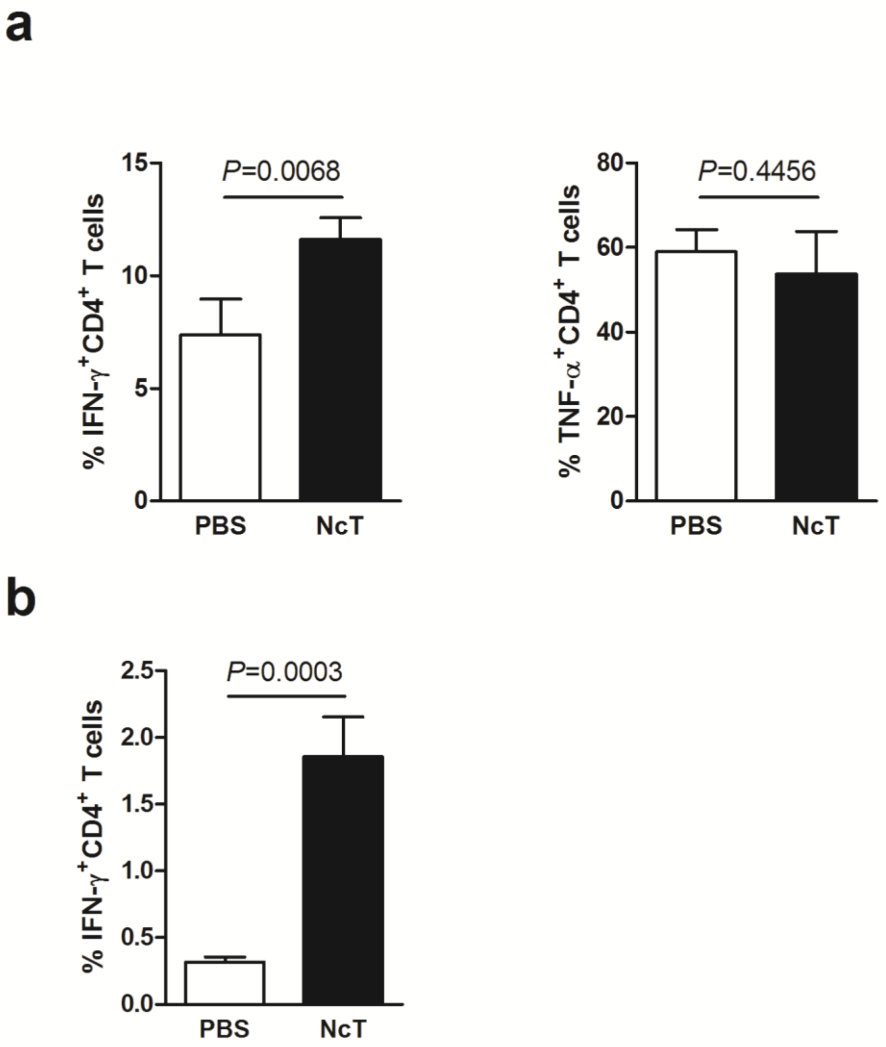

(a) Percentage of CD4-gated T cells expressing IFN-γ or TNF-ɑ in the spleen of *N. caninum* i.p. infected *CD8a*$^{-/-}$ mice (NcT) and sham-infected controls (PBS), detected by intracellular staining after stimulation with PMA/ionomycin 7 days upon the parasitic challenge. (b) Percentage of CD4-gated T cells expressing IFN-γ detected in spleen cell cultures of 7-day infected *CD8a*$^{-/-}$ mice (NcT) and sham-infected controls (PBS), after 16 h *N. caninum* antigen-stimulation. Bars represent the mean plus one SD (n=3 for controls and n=4 for infected mice). Statistical significance between infected mice and controls is indicated above bars.

Supplementary Figure S6: Parasitic load in *N. caninum*-infected C57BL/6 WT, *p47phox*$^{-/-}$ and *Nos2*$^{-/-}$ mice

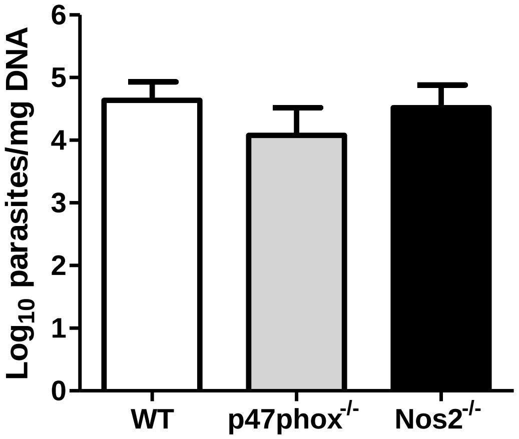

Parasitic load of brain tissue assessed by qPCR specific for *N. caninum* DNA in C57BL/6 WT (n=10), *p47phox*$^{-/-}$ (n=9) and *Nos2*$^{-/-}$ (n=8) mice, as indicated, 30 days after i.p. inoculation with 1×10^7 *N. caninum* tachyzoites. Bars represent means plus one SD. No statistically significant differences were observed among the different infected mouse groups (One-way ANOVA and Tukey's *post-hoc* test).

Supplementary Figure S7: Expression of inducible nitric oxide synthase (*Nos2*) mRNA in infected and control B6 WT and *CD8a*$^{-/-}$ mice

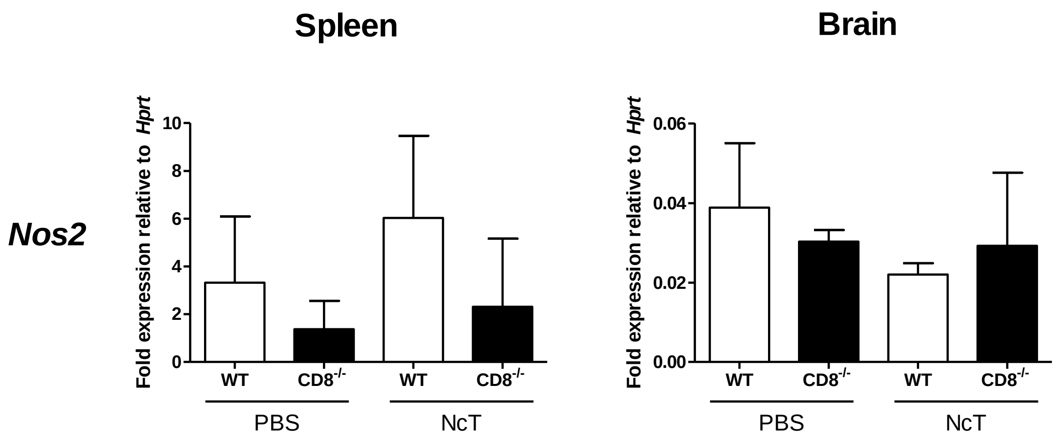

Relative levels of *Nos2* mRNA, normalized to hypoxanthine guanine phosphoribosyl transferase (*Hprt*) mRNA, detected by real-time PCR in the spleen and brain of WT and *CD8a*$^{-/-}$ mice, as indicated, 7 days after i.p. injection of 1×10^7 *N. caninum* tachyzoites (NcT; n=4) or PBS (PBS; n=3). Bars represent mean values of the respective group plus one SD. No statistical significant differences were observed among mouse groups (one-way ANOVA and Tukey's post-hoc test).

Supplementary Figure S8: *Rag2*$^{-/-}$ mice are lethally susceptible to *N. caninum* infection

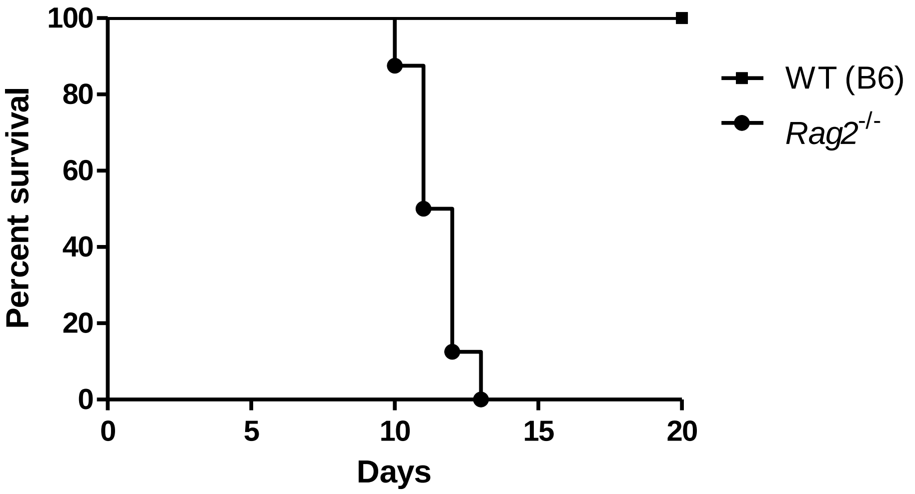

Survival of C57BL/6 WT (n=10) and *Rag2*$^{-/-}$ (n=10) mice infected i.p. with 1×10^7 *N. caninum* tachyzoites. Statistical difference between mouse groups was calculated with the log-Rank test (P<0.001).

Supplementary Figure S9: Expression of IFN-γ by splenic T cells of *N. caninum* infected C57BL/6 WT and *Prf1*$^{-/-}$ mice

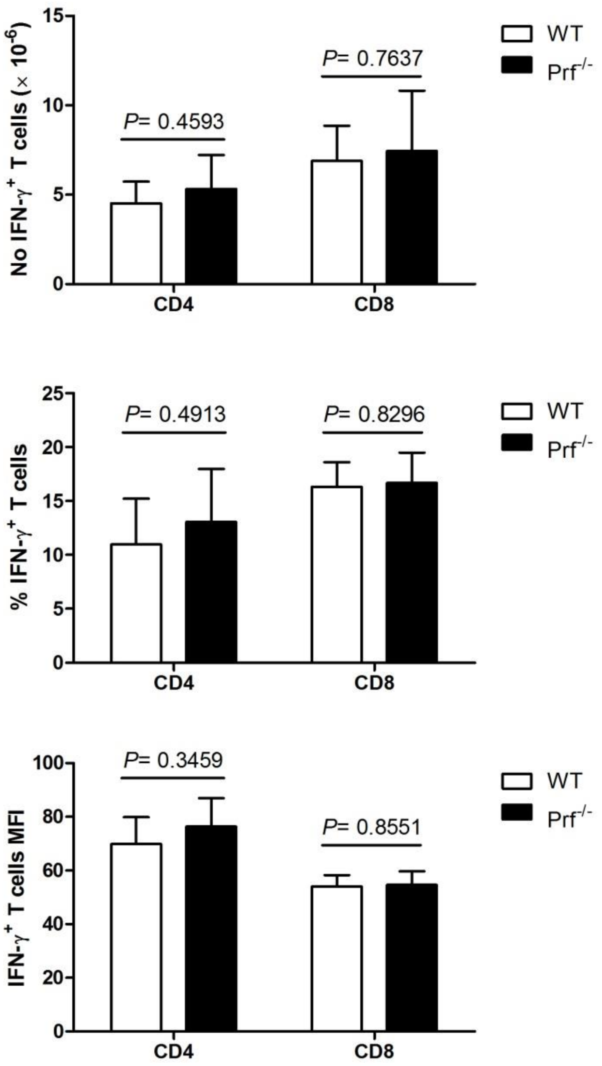

Numbers, proportions and MFI due to IFN-γ staining of IFN-γ-producing CD4$^+$ and CD8$^+$ T cells, as indicated, detected in the spleen of WT and *Prf1*$^{-/-}$ mice 7 days upon infection with 1×10^7 *N. caninum* tachyzoites. Bars represent means plus one SD of five mice per group. Statistical significances between mouse groups are indicated above bars.

Supplementary Table S1: Reconstitution of *Rag2*$^{-/-}$ mice

Relative proportions and total numbers of $CD4^+$ and $CD8^+$ T cells detected by flow cytometry in the spleen of 7-day *N. caninum* infected *Rag2*$^{-/-}$ mice reconstituted 28 days prior to infection with 1.5×10^6 WT $CD4^+$ T cells and equal numbers of either WT or *Ifng*$^{-/-}$ $CD8^+$ T cells, as indicated.

Individual mouse number	*IFNG* gene status in $CD8^+$ T cells	$CD4^+$ T cells		$CD8^+$ T cells	
		%	total number (millions)	%	total number (millions)
#1	-/-	80,07	12,35	19,93	3,07
#2	-/-	86,46	19,82	13,54	3,10
#3	-/-	93,45	18,54	6,55	1,30
#4	-/-	83,38	18,23	16,62	3,63
#5	-/-	83,70	21,46	16,30	4,18
#6	-/-	78,68	33,30	21,32	9,02
#7	-/-	77,65	46,79	22,35	13,47
#8	-/-	90,13	24,53	9,87	2,69
#9	-/-	88,57	31,10	11,43	4,01
#10	-/-	78,93	13,31	21,07	3,55
#11	+/+	82,45	34,89	17,55	7,43
#12	+/+	60,98	38,04	39,02	24,34
#13	+/+	7,26	3,53	92,74	45,05
#14	+/+	74,22	37,33	25,78	12,97
#15	+/+	74,85	44,02	25,15	14,79
#16	+/+	80,69	43,86	19,31	10,49
#17	+/+	72,21	39,36	27,79	15,15
#18	+/+	62,35	48,19	37,65	29,10
#19	+/+	76,88	26,86	23,12	8,08
#20	+/+	85,22	10,48	14,78	1,82
